# Multiple Urethral Stones Causing Penile Gangrene

**DOI:** 10.1155/2014/182094

**Published:** 2014-05-18

**Authors:** Michael J. Ramdass, Vijay Naraynsingh

**Affiliations:** Department of Clinical Surgical Sciences, University of the West Indies, St. Augustine, General Hospital, Port-of-Spain, Trinidad and Tobago

## Abstract

Penile urethral stones are a rare occurrence resulting from a number of causes including migration of stones within the urinary tract, urethral strictures, meatal stenosis, and obstructing tumours such as adenomatous metaplasia of the uroepithelium, hypospadias, urethral diverticulum, and very rarely primary fossa navicularis calculi. We report the case of a 54-year-old male presenting with penile gangrene and sepsis resulting from impaction of multiple stones within the penile urethra. This paper summarises the topic and discusses the pathophysiology of this unusual condition.

## 1. Introduction

Penile urethral stones are a rare occurrence with an incidence of less than 0.3% [1], resulting from a number of causes including migration of stones within the urinary tract, urethral strictures, and obstructing tumours such as adenomatous metaplasia of the uroepithelium, hypospadias, urethral diverticulum, and very rarely primary fossa navicularis calculi. However, its association with penile gangrene is nonexistent in the world literature via a standard PubMed search on the topic. Therefore, to the best of our knowledge we herein report the first documented case of penile urethral stones and its direct association with penile gangrene.

## 2. Case Presentation

A 54-year-old male presented to the Accident and Emergency Department with inability to pass urine and a painful, foul-smelling, and swollen penis for 3 days. He had no known medical problems and was not diabetic. On admission, the skin of the penis was partially necrotic with progressing gangrene, it was malodourous, and the abdominal wall and scrotum appeared normal.

Ultrasound, retrograde urethrogram, intravenous urogram, and urethroscopy were done within 24 hours. These confirmed a normal upper urinary tract and bladder and showed obstruction of the penile urethra secondary to multiple calculi; however, the calculi were embedded and the tissues were grossly inflamed and ulcerated. He was started on intravenous ampicillin, gentamicin, and metronidazole which were continued for 5 days during his hospital stay followed by amoxicillin/clavulanic acid upon discharge for a further week.

**Figure 1:**
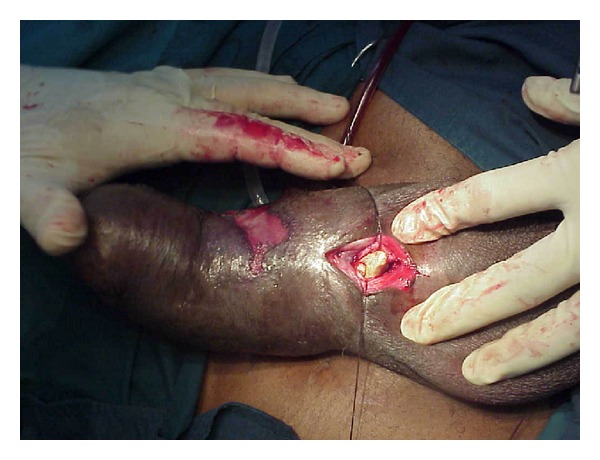
Urethral stone exposed via ventral approach.

**Figure 2:**
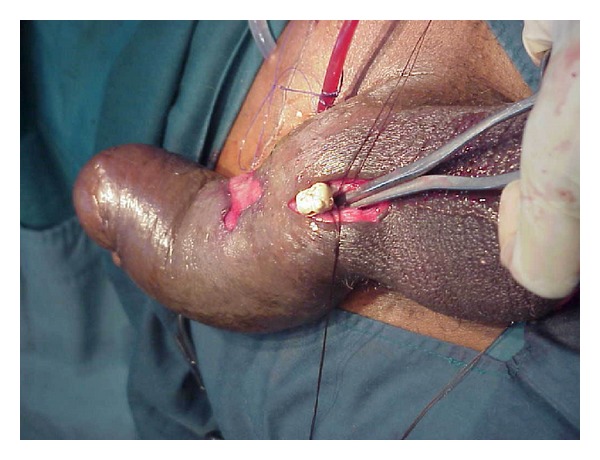
Stone being extracted using an artery forceps.

**Figure 3:**
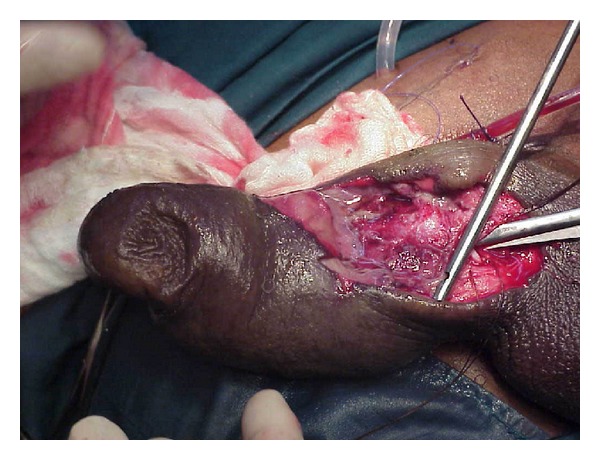
Necrotic area of penis opened and debrided.

**Figure 4:**
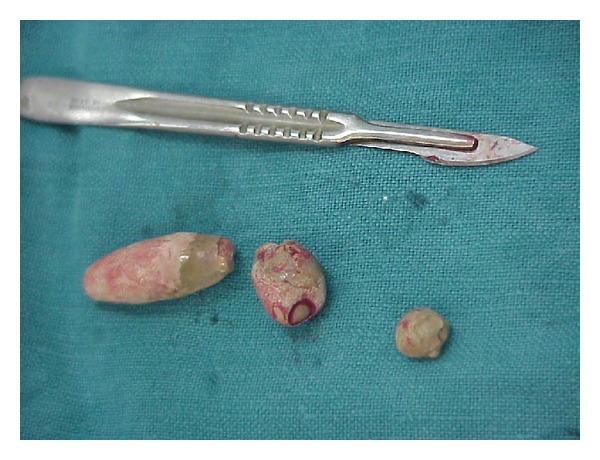
Multiple extracted urethral stones.

The patient consented for extraction of the urethral stones as well as aggressive debridement of the penile tissues with the risk of amputation. The exploration was done under general anaesthesia and an incision was done on the ventral (urethral) surface of the penis. Through a ventral urethrotomy, three urethral stones were successfully removed and the soft tissues of the penis aggressively debrided. A suprapubic cystostomy was also done and a catheter was inserted at this level (see Figures 1, 2, 3, and 4). The ventral penile urethrotomy was closed primarily over a long-term catheter and the penile wounds were dressed and later closed with a scrotal skin advancement flap. Circumcision was not done as the skin though swollen was viable, and having already lost some penile skin to gangrene, all the intact remaining skin was conserved. The preputial oedema settled within 5 days of debridement. The urethral catheter was removed at 4 weeks and the suprapubic catheter was removed at 6 weeks. On a 12-month follow-up he had no evidence of erectile dysfunction or urethral stricture.

## 3. Discussion

Penile urethral calculus is a rare form of urolithiasis with an incidence lower than 0.3%. Patients may present with acute urinary retention, interrupted or weak stream, gross haematuria, or pain affecting the penis, urethra, or perineum.

Etiological factors include a range of pathologies including the migration of calculi originating higher up in the urinary tract, penile urethral strictures which are strongly associated with chronic infections of the urethra [2, 3], tumours of the urethra such as adenomatous metaplasia of the uroepithelium [4], or meatal stenoses that may occur in young boys [5]. There may also be an association with the presence of a urethral diverticulum [6] which has been documented in a single case by Shiraishi et al. in 1989 in a 54-year-old female.

Even rarer associations have been documented whereby urethral calculi form within the fossa navicularis which is the dilated portion of the male urethra just distal to the narrower cavernous portion [1]. It is also known as the spongy part of the male urethra and lies within the glans area.

In an analysis done by Verit et al. in 2006 there were fifteen patients studied, eight of which were paediatric cases affecting the fossa navicularis. The stones were all fusiform in shape and solitary. Six of these also had urethral pathologies including hypospadias and one case with a diverticulum.

The patient herein presented in this case report had clinical features of anuria, penile and perineal pain, and penile gangrene due to obstruction. There was a chronic inflammatory process at the time and quite possibly in the weeks leading up to the acute event. The stones at the time of surgery were embedded in the urethra making it difficult and near impossible to be removed via standard minimally invasive techniques such as urethroscopy.

This case highlights two main points, the first emphasizing that there is still a place for performing open operations especially in this difficult situation with chronic, impacted, and multiple stones as well as being able to efficiently debride and control sepsis and the second noting that this association with gangrene and urethral stones has not been previously documented clearly in the surgical literature. A standard search of the medical literature on PubMed reveals no article associating penile gangrene with urethral stone disease to the best of our knowledge. We hope this case is useful for our colleagues practising in the field of emergency medicine, nursing and urology and brings attention to this particular condition and its management.
